# Supplementary material for: The Prognostic Impact of ABO Blood Group in Hepatocellular Carcinoma Following Hepatectomy
Source: Cancers (Basel). 2023 May 25;15(11):2905. doi: 10.3390/cancers15112905 (PMC10251914; doi:10.3390/cancers15112905)
Supplement: Supplementary file 1 [file cancers-15-02905-s001.zip › cancers-2339450-supplementary.pdf]

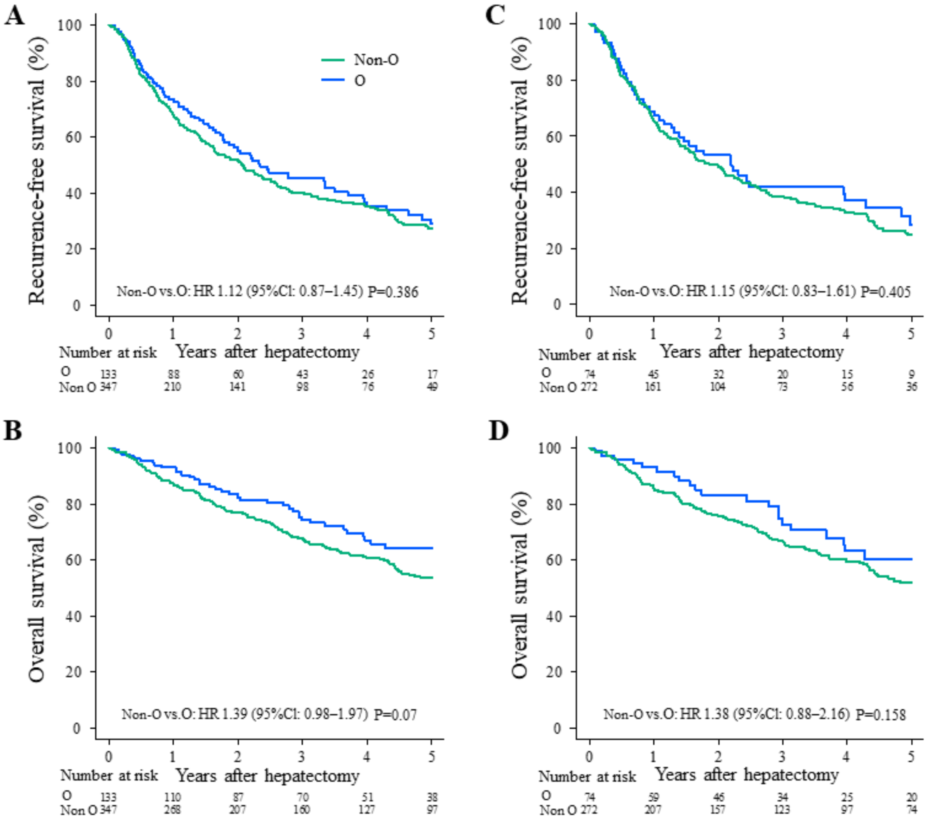

Figure S1. Survival outcomes. Comparison of survival outcomes after hepatic resection for type O (blue) and non-type O (green) patients. (A) RFS and (B) OS before propensity score matching. (C) RFS and (D) OS after propensity score matching. HR, hazard ratio; CI, confidence interval.
